# Stem Endophytic Mycobiota in Wild and Domesticated Wheat: Structural Differences and Hidden Resources for Wheat Improvement

**DOI:** 10.3390/jof6030180

**Published:** 2020-09-18

**Authors:** Xiang Sun, Evsey Kosman, Amir Sharon

**Affiliations:** Institute for Cereal Crops Improvement, School of Plant Sciences and Food Security, Faculty of Life Sciences, Tel-Aviv University, Tel-Aviv 6997, Israel; sunx@tauex.tau.ac.il (X.S.); kosman@tauex.tau.ac.il (E.K.)

**Keywords:** fungal endophytes, wheat, wild wheat, *Aegilops*, mycobiome

## Abstract

Towards the identification of entophytic fungal taxa with potential for crop improvement, we characterized and compared fungal endophyte communities (FECs) from domesticated bread wheat and two wheat ancestors, *Aegilops* *sharonensis* and *Triticum* *dicoccoides*. Data generated by next generation sequencing identified a total of 1666 taxa. The FECs in the three plant species contained high proportions of random taxa with low abundance. At plant species level, the majority of abundant taxa were common to all host plants, and the collective FECs of each of the three plant species had similar diversity. However, FECs from the wild plants in specific sites were more diverse and had greater richness than wheat FECs from corresponding specific fields. The wild plants also had higher numbers of differentially abundant fungal taxa than wheat, with *Alternaria infectoria* being the most abundant species in wild plants and *Candida sake* the most abundant in wheat. Network analysis on co-occurrence association revealed a small number of taxa with a relatively high number of co-occurrence associations, which might be important in community assembly. Our results show that the actual endophytic cargo in cultivated wheat plants is limited relative to wild plants, and highlight putative functional and hub fungal taxa with potential for wheat improvement.

## 1. Introduction

Terrestrial plants contain communities of fungal endophytes that occupy all plant parts, from roots to seeds. In general, the vast majority of the taxa in a given fungal endophyte community (FEC) are sporadic, and only a relatively small set of more predominant taxa show association with specific factors, such as host genotype, environmental conditions, or plant organs [1,2]. It is also evident that the communities are highly stochastic and variable, and include a large proportion of rare taxa [3]. A small number of fungal genera, mainly *Epichloë* and *Piriformospora,* include specific mutualistic species [4,5], but the vast majority of fungal endophytes are still defined as commensals because they have no obvious impact on their hosts [6,7]. In this respect, it is important to distinguish between sporadic and rare taxa, and the more stable and predominant taxa, which might be part of the core microbiome. Generally, core taxa are considered of functional importance, either by directly affecting the host [8,9], or by shaping the assembly of plant-associated microbiomes [10,11]. With the accelerated accumulation of next generation sequencing (NGS) data on plant microbiomes, the challenges ahead are to define core versus sporadic taxa [12], differentiate between mutualists and commensals [13], and identify hub taxa and functional species [11,14].

Wild plants are far more diverse and are grown under a much wider range of conditions than crop plants, entailing that FECs in wild species might be enriched for beneficial taxa. For example, FECs from bread wheat (*Triticum aestivum*) were less diverse than FECs from the closely related *Triticum urartu* [15]. To fully evaluate the true potential of wild species FECs for crop improvement, it is necessary to analyze FECs in population of wild species and compare them with those of related crops. Such analyses will contribute to better evaluation of the natural diversity of FECs in wild plant species, determination of what part of the diversity might be missing in related crops, and helpuncover functional core components and potentially functional sub-groups.

In this study, we analyzed and compared FECs in stems of the hexaploid bread wheat *T. aestivum* L., and two related wild species: *Triticum dicoccoides* Koern. (wild emmer wheat), a tetraploid species that is the ancestor of domesticated durum wheat, and *Aegilops sharonensis* Eig (sharon goatgrass), a diploid species of the section Sitopsis. Samples of the two wild species were collected at their center of origin around Israel from natural populations growing alongside cultivated wheat fields. We show that the FECs in wheat related wild plants contain a richer repertoire of taxa and are more diverse than the FECs in cultivated wheat. Diversity and network analyses revealed a small number of core and hub taxa, which might affect plant performances and play a role in FEC assembly, respectively.

## 2. Materials and Methods

### 2.1. Plant Material

**Figure 1:**
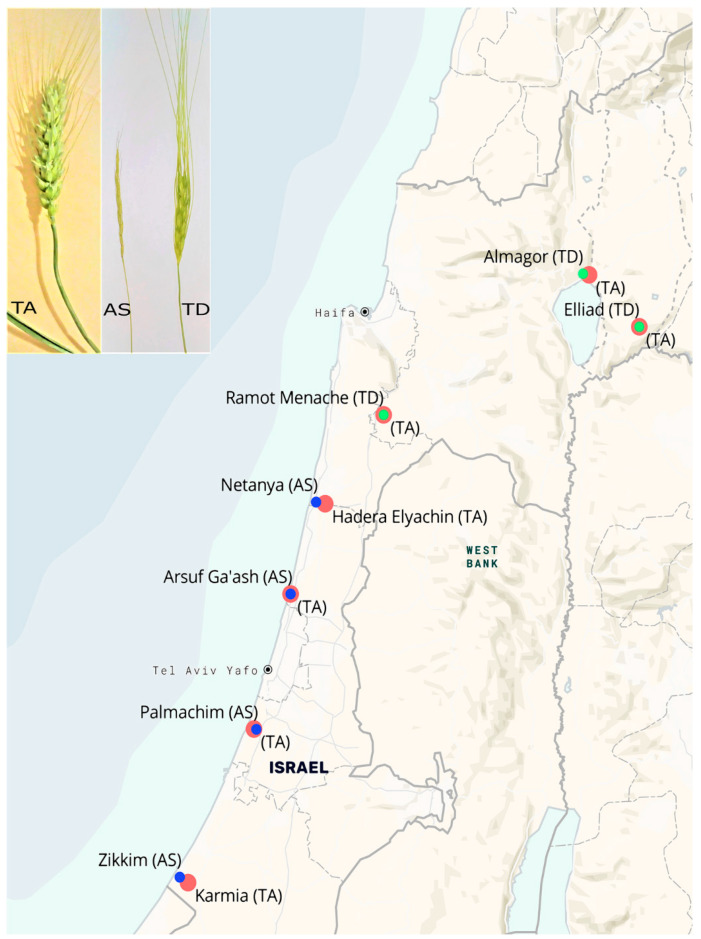
Collection sites of *Triticum aestivum* (TA, wheat), *Aegilops sharonensis* (AS, sharon goatgrass) and *Triticum dicoccoides* (TD, wild wheat). *A. sharonensis* is an endemic species that grows only in a specific region along the Mediterranean coast of Israel, *T. dicoccoides* grows in specific habitats in the northern parts of the country. Locations of paired sampling: TA/AS—blue circles, TA/TD—red circles.

**Collection sites:** Plants were collected from seven sites within the boundaries of Israel (Table 1) during March and April 2017. In all cases, the plants were collected after heading and before maturation. Sharon goatgrass (*A*. *sharonensis*) plants were collected from four sites, and wild wheat (*T*. *dicoccoides*) plants were collected in three sites. Wheat (*T. aestivum*) plants were collected from seven commercial fields, each located near one of the seven sites from which wild plants were collected (Figure 1). The plants were collected using a random sampling approach from a 50 × 50 m^2^ quadrant plot in each site.

**Sample preparation**. Plants were uprooted with soil, placed in a 4 °C container, and processed within 24 h. Stems segments were cut, surface-sterilized by submerging in 0.5% commercial bleach for 2 min, and washed by dipping three times in sterilized distilled water. The sterilized stem samples were cut into five mm pieces, and 150 mg samples that included pieces from each intermodal segment were placed in Eppendorf tubes, snap frozen in liquid nitrogen, and stored at −80 °C freezer.

### 2.2. Extraction of Genomic DNA

DNA was extracted as described by Sun et al. [2]. In brief, samples were lyophilized overnight and homogenized to a fine powder using a Geno/Grinder 2000 (OPS Diagnostics, Bridgewater, NJ, USA). DNA was extracted by adding to each homogenized sample 1 mL of CTAB extraction buffer supplemented with 50 µg/mL of proteinase-K. DNA integrity was checked by agarose gel electrophoresis and quantified by Picogreen assay (Thermo Fischer Scientific Inc., Waltham, MA, USA).

### 2.3. Amplicon Library Preparation and Sequencing

ITS amplicon libraries were produced from 596 DNA samples by the Environmental Sample Preparation and Sequencing Facility (ESPSF) at Argonne National Laboratory (as described by Sun et al. [2]). Briefly, amplicon libraries targeting the fungal ribosomal ITS1 region were produced using ITS1f (5′-CTTGGTCATTTAGAGGAAGTAA-3′) and ITS2r (5′-GCTGCGTTCTTCATCGATGC-3′) primers [16]. The PCR reaction was supplemented with a custom PNA blocker (ITS1PNABlk: 5′-O-E-E-GTCGTGTGGATTAAA-3′) (PNA BIO INC, Thousand Oaks, CA 91320, USA) that was designed to prevent amplification of host plant ITS sequences. Equimolar volumes of amplicon libraries were pooled and quantified, the samples were diluted to a final concentration of 6.75 pM, and then sequenced on MiSeq run of 251 cycles forward read, 12bp index for barcodes, and 251 cycles reverse read, using customized sequencing primers and procedures [17]. All the sequencing data were deposited in NCBI Small Read Archive (SRA) with the accession number PRJNA509176 and the bioproject number PRJNA509176.

### 2.4. Data Analyses

#### 2.4.1. Quality Control and Data Preparation

Initially, forward and reverse reads were demultiplexed and quality control check was performed in QIIME2 platform version 2018.11 [18]. Reverse primers occurring in their reverse complement form at the tail-end of the forward reads were trimmed using the Cutadapt tool [19]. Subsequently, the DADA2 workflow [20] was used for quality filtering, dereplication and sample inference. Following quality control, the reverse reads were excluded, as they invariably showed poor quality. Following the quality control, 29 samples were eliminated, and amplicon reads in the remaining 567 samples were dereplicated to form unique sequences. The average of the positional qualities from the dereplicated reads was retained, as the consensus quality profiles of unique sequences to inform the error model of the subsequent pooled sample inference step, thereby increasing the accuracy of the DADA2 algorithm. In total, 1,801,550,768 nucleotide bases in 8,822,899 forward reads were used for learning the error rates and sample inference, based on the consensus quality profiles of unique sequences to infer 2823 exact amplicon sequence variants (ASVs). Of the 2823 ASVs, 268 chimeric amplicon sequences were identified using the “removeBimeradenovo” option in DADA2 workflow, and these sequences were discarded. In addition, reference-based chimera removal approach was used to remove chimeric fungal ITS1 sequences. Subsequent BLAST analyses revealed that a certain proportion of the chimeric sequences flagged by the reference-based approach showed a highly significant match to fungal UNITE database, and hence these sequences were not filtered. To remove host ITS sequences, we performed BLAST analysis of the ASVs against wheat ITS dataset, with the following threshold values: 1e−50, percentage similarity value >97%, and percentage query coverage >95%. A total of 96 host plant ITS sequences were removed by this procedure. After dereplicating the redundant reads and removing chimeric reads, unique sequences were binned into 2450 ASVs. Taxonomic assignment was performed using the naïve Bayes approach, with a minimum of 50 bootstrap calls against the UNITE fungal reference dataset [21], and ASVs that were assigned to plants and other contaminants were removed from the final ASV table (Table S1). Following the initial taxonomic analysis of the final set of ASVs, we agglomerated ASVs with identical taxonomic assignments at the species level and removed samples with sparse reads (less than 1000), which yielded 1687 taxa (including species from agglomeration and ASVs taxonomically unassigned at species level) across 530 samples. With singletons and doubletons removed, 1666 taxa remained in the dataset (Table S2). For beta diversity and differential abundance analyses, ASVs with less than three reads and presence in less than five samples were further removed (Table S3).

#### 2.4.2. Variation within FECs

The set of ASV reads was Hellinger transformed for analysis of the abundance data. Most abundant taxa were inferred by the “ampvis2” package [22]. A boxplot was generated using *amp_bpxplot* function, wherein the top 18 fungal taxa were ordered by median read abundance values across all samples. The fungal ASV richness per individual plant and average ASV richness per plant species were calculated using *specnumber* function of “vegan” package [23]. Since the observed species richness is often lower than the true species richness [24], singletons and doubletons were not filtered for measuring alpha diversity [25]. Within FEC diversity was estimated for incidence data with Hill numbers for *q* = 0, 1, 2 (species richness, the exponential of Shannon entropy, and the inverse of Simpson concentration, respectively) using the “iNEXT” package [26].

#### 2.4.3. Variation among FECs of Separate Plants within a Population

The Bray–Curtis dissimilarity [27] and its binary analog, Dice dissimilarity [28] were used for measuring pairwise dissimilarities for the abundance and incidence data, respectively. Variation within each population was estimated based on the incidence data. First, the assignment based dispersion *KW* [29,30,31] with regard to the Dice dissimilarity was calculated. Then, the effective number of different FECs (plants) and its normalized version ${}_{\;}^{1}nD\left(T,KW\right)$ (Equation (3) in Sun et al. [2]) were estimated for each species in different locations, and for the entire set of all FECs (plants) of a given species.

#### 2.4.4. Variation among FECs of Host Species and Locations 

Differentiation analyses were performed, based on both qualitative (incidence data) and quantitative (Hellinger transformed abundance data) compositions of the FECs for each plant. The principal coordinate analysis (PCoA) with regard to the Dice and Bray–Curtis dissimilarity matrices was used to establish relationships of fungal communities of different species. A constrained analysis of principal coordinates (CAP) was utilized to explore the relationship between fungal communities and host genotype. Permutational multivariate analysis of variance (PERMANOVA) was used to test an effect of host genotypes and geographic locations on differentiation among the corresponding FECs and overall beta diversity [32]. 

Variation of FECs among populations of the same host from different locations was evaluated based on Kosman’s distance between populations and the differentiation statistics *D*, assuming the additive partition of the average-based dispersion *M* of Kosman’s distances; the estimate *D* was tested for significance [2,29,33]. The effective number ${}_{\;}^{1}D\left(TM\right)$ of different populations (individual FECs of plants within each population in a given set) and its normalized version ${}_{\;}^{1}nD\left(TM\right)$ were calculated according to Scheiner et al. [34] and Equation (3) in Sun et al. [2]) (corrected Equation (5) in Kosman et al. [35]), respectively. 

To make a statistically robust prediction of differentially abundant taxa, and to account for variable library sizes, the differential enrichments of fungal taxa between *T. aestivum* and *T*. *dicoccoides*, and between *T*. *aestivum* and *Ae. sharonensis*, were evaluated with *DESeq* function in “DESeq2” package [36], which take sizes and dispersion of data into consideration.

#### 2.4.5. Co-Occurrence Analysis

Co-occurrence association among endophytic taxa were evaluated and Spearman’s *rho* were calculated with *cor.test* function in “stats” package [37]. The analyses were applied at genus level of fungal taxa, and the taxa that failed to be assigned to a genus were removed. The associations were calculated for each population (all plant individuals of a host from a specific site), and then the co-occurrence association sets of a plant species were combined. Then, the count of a certain co-occurrence association (namely, edges in network when visualized with graphs) present in all populations was recorded in the combined association sets. In addition, incidence of each associated pairwise among sites and its accumulated *rho* (for positive and negative values, respectively) were also recorded. Considering the high stochasiticity in endophytic fungal communities of wild cereals [2], we assumed that the associated pairwise, which occurred in only one population, were probably caused by random factors and thus discarded them. Co-occurrence association networks visualized for each host combining all associated pairwise events that occurred at least twice. In addition, the nodes with highest degree and closeness centrality, and lowest betweenness centrality, were considered as keystone or hub fungal taxa [38].

## 3. Results

### 3.1. Composition and Taxonomy of the Fungal Endophytes

**Figure 2:**
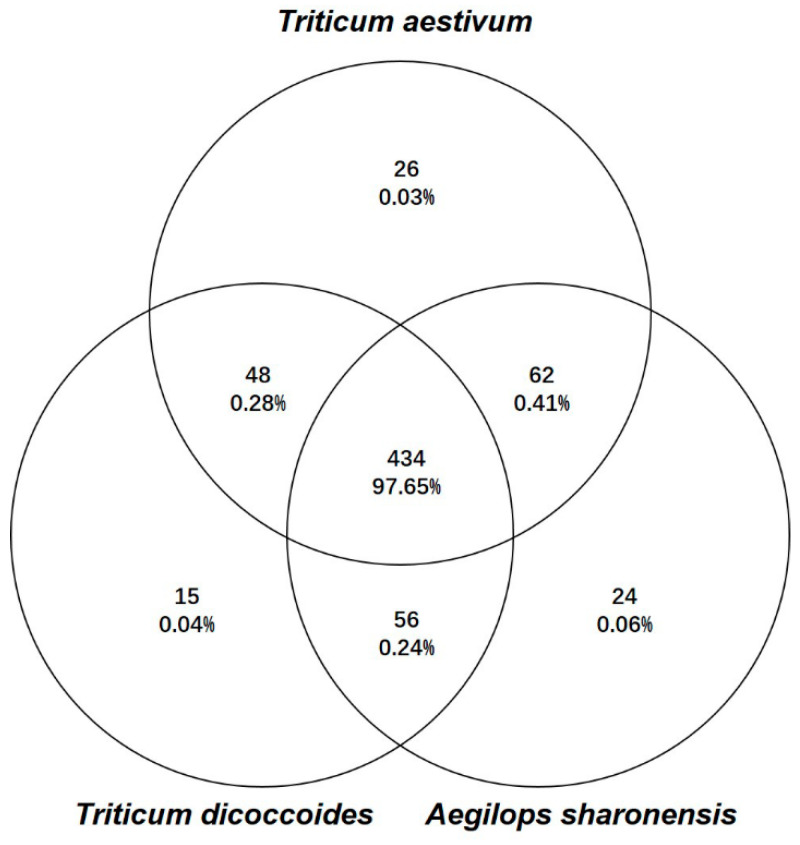
Shared and unique fungal taxa among the fungal endophyte communities (FECs) of the three plant species. The Venn diagram shows 665 taxa in core dataset with prevalence threshold of >5. Percentage indicates relative abundance of corresponding group of taxa within the overall dataset covering all 1666 agglomerated taxa. In total, 1001 taxa that account for 1.28% of the reads did not meet the prevalence threshold and were removed from the core dataset.

**Figure 3:**
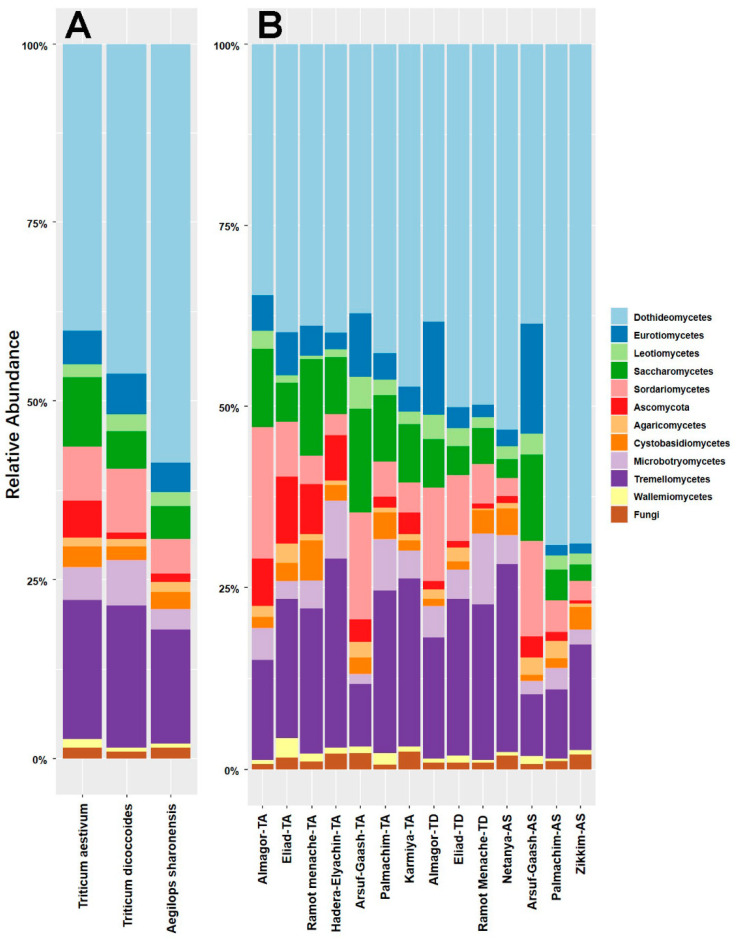
Relative abundance of fungal classes in each of the 14 plant populations. The stacked bar plot depicts top 12 fungal classes based on relative abundances and assorted according to the host species (**A**) and populations (**B**).

The dataset of 1687 taxa across 530 samples was filtered to remove singletons and doubletons, resulting in a final dataset of 1666 taxa in 530 samples. We further removed rare taxa with incidence <5 (1001 taxa, 1.28% of total abundance), resulting in a core dataset of 665 taxa across 530 samples. In this core dataset, 396 taxa were assigned to 159 genera, in which 253 taxa were assigned at species level. In addition, 434 taxa, which accounted for 97.65% of the reads, were found in all three plant species (Figure 2). *T*. *aestivum, Ae. sharonensis,* and *T. dicoccoides* each contained 570, 576, and 553 taxa with 26, 24, and 15 unique ones, respectively. The host-specific taxa had low abundance and collectively accounted for only 0.13% of the reads. Therefore, the core set of FECs showed a generally overlapping pattern. The vast majority of fungal taxa were assigned to the phyla Ascomycota (69.47% of total taxa) and Basidiomycota (21.50%). Among the remaining taxa, five were assigned to *Mucoromycota* and *Mortillomycota*, and 55 taxa could not be assigned at phylum level. At class level, *Dothiomycetes* were the dominant class in all three plant species, followed by *Tremellomycetes* and *Sordariomycetes* (Figure 3), while taxa belonging to the family *Pleosporaceae* were the most abundant taxa in FECs from the two wild plant species, but not in wheat (Figure S1). Compared to *Ae. sharonensis* and *T. dicoccoides*, the wheat FECs had less abundant Dothideomycetes and more abundant Saccharomycetes and unassigned. 

**Figure 4:**
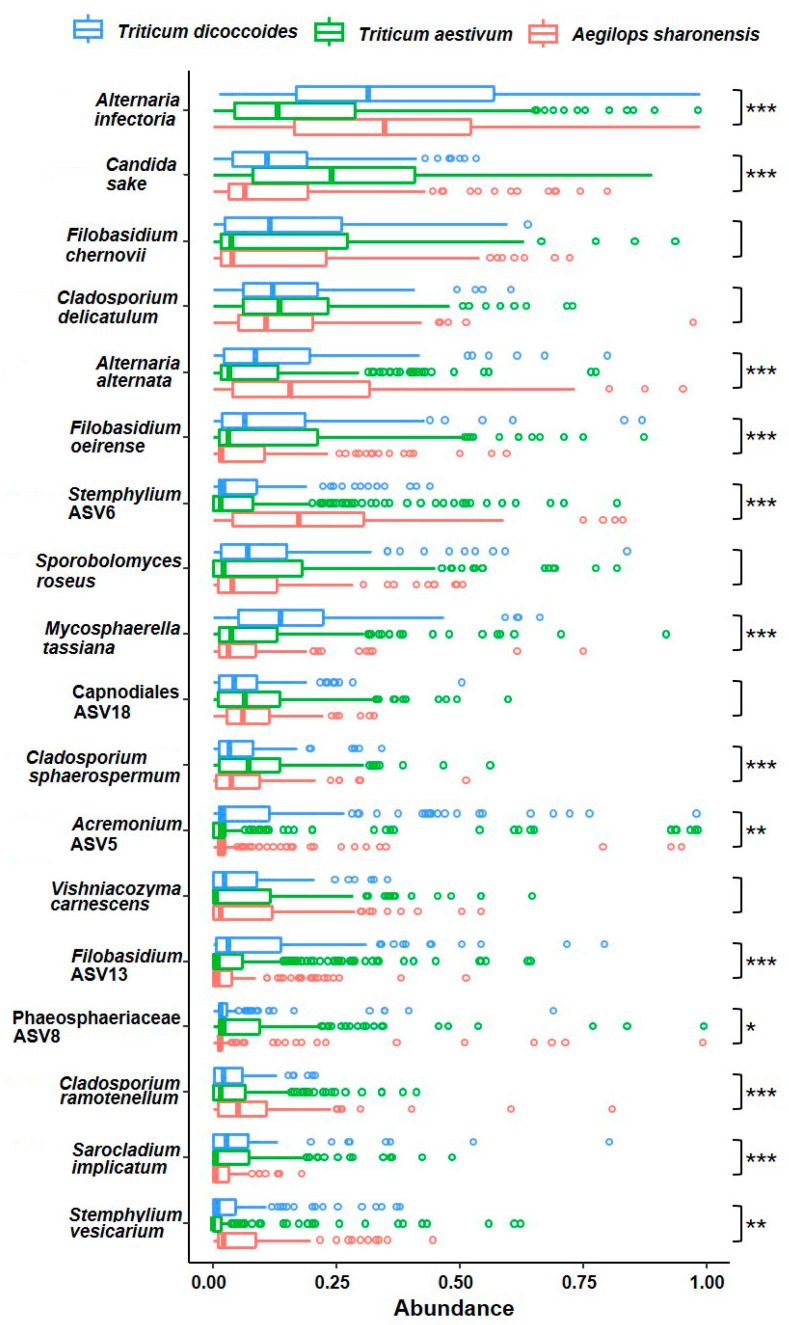
Abundance of the most dominant fungal taxa in each of the three plant species. The top 18 most abundant endophytic taxa accounted for 50% of total abundance in the core dataset. The Hellinger transformed abundance of each taxon is shown for each of the three host plants. Outliers are plotted with open cycles. One-way ANOVA was used to test the significance of abundance difference among hosts (*p* < 0.001, ***; *p* < 0.01, **; *p* < 0.05, *).

The most abundant species in the entire population was *Alternaria infectoria*, followed by *Candida sake, Filobasidium chernovii*, *Cladosporium delicatulum*, and *Alternaria alternata*, but the relative abundance of certain taxa varied in FECs from different plant species (Figure 4). For example, *A. infectoria* was the most abundant species in the FECs from the two wild plant species, while *C. sake* was the most abundant species in FECs from wheat. Similarly, *C. sphaerospermum*, and Phaeosphaeriaceae ASV8 were relatively more abundant in FECs from wheat than in *T. dicoccoides* and *Ae. sharonensis* FECs, whereas *A. alternata*, *C. ramotenellum*, *Stemphylium vesicarium*, and *Stemphylium* ASV6 were more highly abundant in FECs from the two wild plant species compared with wheat FECs. Collectively, the taxonomic analyses show that wheat FECs differ from those of the wild species.

### 3.2. Effect of Plant Species on Variation within FECs

**Figure 5:**
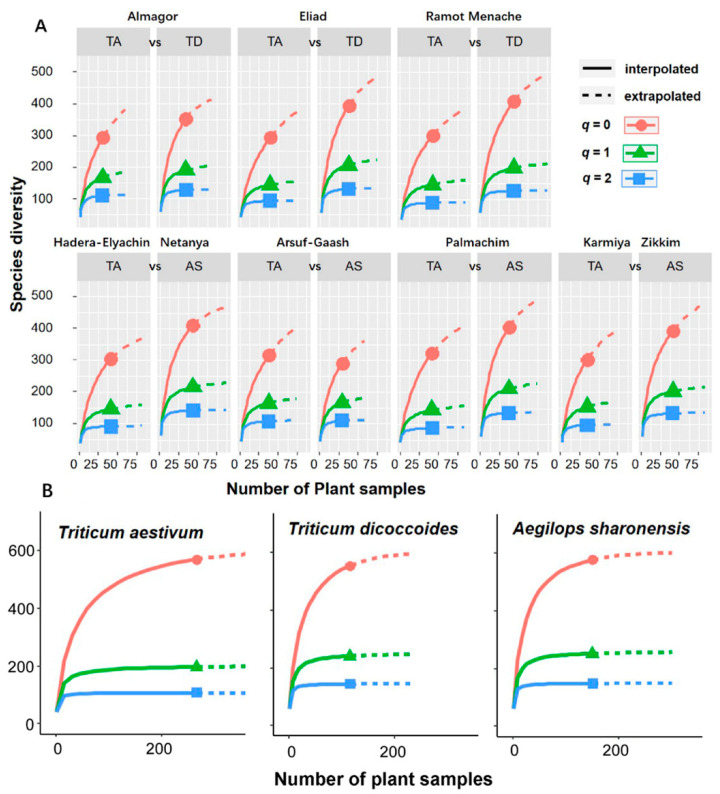
FECs from the wild plant species are richer and more diverse that wheat FECs. The sample-size based rarefaction and extrapolation of Hill numbers of order *q* are shown for the FECs in each of the 14 sites (**A**) and for pooled populations of each of the host plants (**B**). TA—*T. aestivum*, AS—*Ae. sharonensis,* TD—*T. dicoccoides*. Red—richness (*q* = 0), Green—exponential of Shannon entropy (*q* = 1), Blue—inverse of Simpson concentration (*q* = 2).

Initial estimates of taxon counts per sample provided a preliminary view on variation within fungal communities in each of the three plant species. The two wild plant species contained a higher number of different fungal taxa than wheat, with an average number of 59 and 58 taxa per plant in *Ae. sharonensis* and *T. dicoccoides*, respectively, compared with 41 taxa per plant in wheat. Diversity of endophytes communities of separate species in different locations was measured with Hill numbers for *q* = 0 (taxa richness), *q* = 1 (rare and prevalent taxa for the incidence data are equally weighted), and *q* = 2 (prevalent taxa are weighted stronger). Rarefaction estimators were applied to avoid bias due to unequal sample sizes. In general, fungal communities from the wild plants showed higher Hill’s values, indicating greater species richness and diversity (Figure 5A, Table 2). Taxa richness of bulk of FECs within a separate location (*q* = 0) in *T. aestivum* was the lowest, and ranged between 292 to 325 taxa per site. Except for a single population (*Ae. sharonensis* from Arsuf-Gaash), the FECs from wild plants had a significantly higher number of taxa within a location than wheat FECs, which ranged between 395–411 taxa in *Ae. sharonensis* and 351–408 taxa in *T. dicoccoides* (Figure 5A). Similarly, the effective number of common taxa (*q* =1) and the effective number of dominant taxa (*q* = 2) were lowest across all FECs from wheat. Thus, irrespective of the geographical location, the FEC diversities of the wild plant species were always higher than those for wheat. The non-overlapping bootstrap confidence intervals suggested that the common (*q* = 1) and dominant (*q* = 2) ASV diversities are different among the three plant species. In addition, asymptotic estimators (Figure 5B) indicate that FECs in the wild plant species are similar with wheat, despite the fact that the FECs from the wild species had higher diversity than those of wheat at most sites.

### 3.3. Variation among FECs of Individual Plants within a Population

Estimates of the normalized variation among FECs of separate populations for the incidence data are shown in Table 3. The normalized variation in *T. aestivum* from each location were higher or similar compared to the variation in *T. dicoccoides*, but smaller or similar in comparison with *Ae. sharonensis*. Nevertheless, the overall variation across different sites of *T. aestivum* is higher than the variations in *Ae. sharonensis* and *T. dicoccoides*.

### 3.4. Effect of Species on FEC Structural Variation among the Populations of Plants

**Figure 6:**
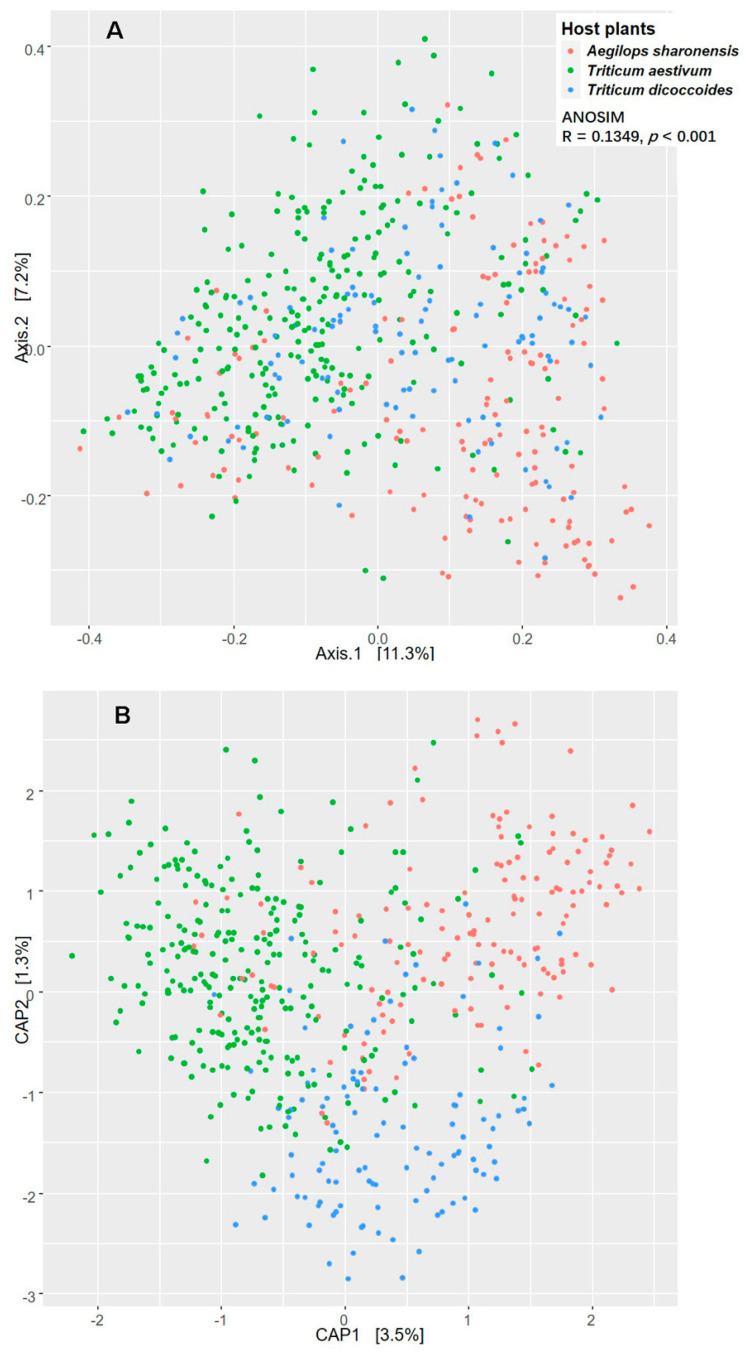
Assessment of the effect of host species on the structure of FECs by PCoA (**A**) and CAP (**B**). The PCoA and CAP ordination based on Bray–Curtis dissimilarity metric were calculated with Hellinger transformed abundance data. ANOSIM shows significance of differentiation among FECs of different species.

Ordination analyses were used to decipher the possible influence of the three plant species on the structure of FEC compositions across the seven geographical locations. PCoA for abundance (Bray–Curtis dissimilarities) and incidence (Dice dissimilarities) showed clear differentiation among FECs from the different plant species (Figure 6A, Figure S1A). CAP plot showed clearly separated communities by hosts (ANOVA: F = 13.17, *p* < 0.001), although the small effect on CAP axis suggested that the host had a relatively small effect on FEC structure (Figure 6B, Figure S1B). ANOSIM analysis based on both abundance and incidence datasets revealed significant differences among hosts and sampling sites. PERMANOVA analysis with Bray–Curtis dissimilarity matrix confirmed that both the plant species and sampling sites had a significant effect on the compositions of FECs (sites: F = 11.07, R^2^ = 0.11, *p* < 0.001; hosts: F = 15.74, R^2^ = 0.05, *p* < 0.001). Similar results were obtained with the Dice dissimilarity matrix (sites: F = 8.23, R^2^ = 0.08, *p* < 0.001; hosts: F = 16.18, R^2^ = 0.05, *p* < 0.001).

The dispersion of FECs compositions of the same species was evaluated, and the extent of differentiation among populations was higher among the *T. aestivum* FECs than FECs from *Ae. sharonensis* or *T. dicoccoides* (Table 4 for incidence data, Table 5 for abundance data). The normalized number of effectively different populations of FECs (plants) of each species was nearly the same for *T. aestivum, Ae. sharonensis* and *T. dicoccoides*. Collectively, these results show a clear effect of the plant species and geographical location on FECs composition.

### 3.5. Co-Occurrence Network Analysis and Identification of Putative Hub Fungal Taxa

**Figure 7:**
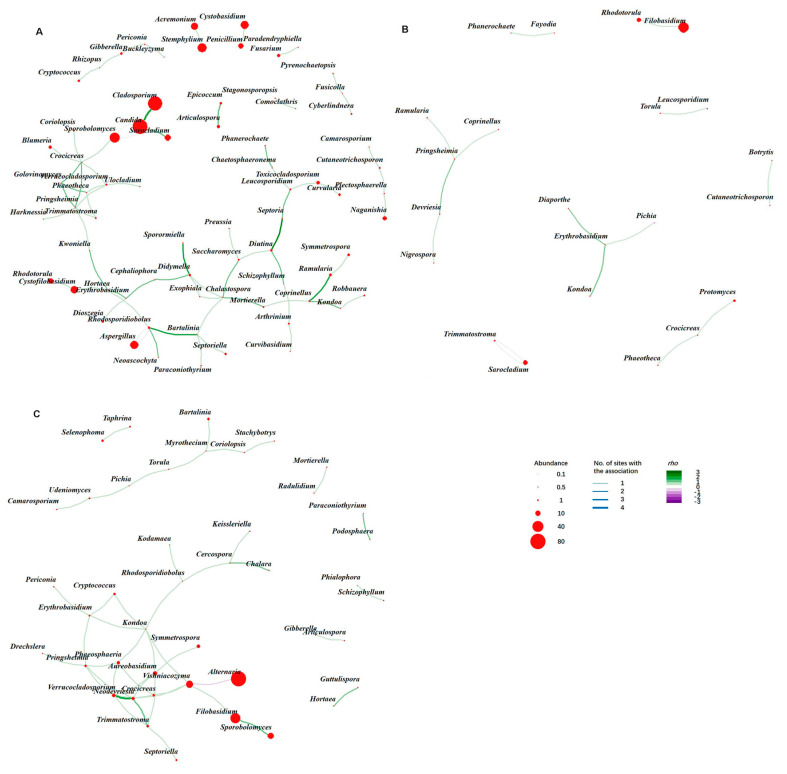
Combined co-occurrence network of FECs in different host species. The combined network includes co-occurrence associations (edges) with statistical significance (*p* < 0.001) presented in no less than two sites in *T. aestivum*—TA (**A**), *T. dicoccoides*—TD (**B**), and *Ae. sharonensis*—AS (**C**). Thicker edges indicate presence of the association in higher number of sites, deeper color indicates higher positive *rho* (green) or lower negative *rho* (purple) for the association.

Combined co-occurrence networks were performed for FECs from each of the three plant species, with the co-occurrence association present in only one site (Figure 7). The network of *T. aestivum* was generated from seven sites, therefore it accumulated more vertices and edges than the networks from *Ae. sharonensis* and *T. diccocides* with four and three sites, respectively (Table S4). Network characteristics such as network diameter, mean distance and edge density of three hosts are shown in Table S4.

Most dominant fungal genera with high abundance, such as *Alternaria*, *Filobasidium*, *Cladosporium*, *Candida*, *Stemphylium*, *Vishniacozyma*, etc., were connected in the co-occurrence network. Nevertheless, the high abundances did not suggest high degrees and eigenvector centralities for the genus. In *T. aestivum*, the genera *Pringsheimia* (Dothideales, Dothideomycetes) and *Crocicreas* (Helotiales, Leotiomycetes) had the highest eigenvector centrality and degree (number of connected taxa) (Figure 7A, Table S5). Additionally, *Verrucocladosporium* (Capnodiales, Dothideomycetes), *Golovinomyces* (Erysiphales, Leotiomycetes), and *Phaeotheca* (Capnodiales, Dothideomycetes) all had high eigenvector centralities and degrees. These genera formed an inter-fungal class module, hinting to a possible importance in assembly of FECs in *T. aestivum*. The *T. diccocides* network was scattered, and no clear core could be observed (Figure 7B, Table S5), possibly due to a low number of sampling sites. *Pringsheimia* and *Erythrobasidium* (Erythrobasidiales, Pucciniomycotina) possessed the highest degrees, although the modules they belonged to did not connect to each other. The *Ae. sharonensis* network had fewer vertices and edges than *T. aestivum* networks, however with a more connected topology (Figure 7C, Table S5). Vertices with high degrees and eigenvector centralities in *Ae. sharonensis* network included *Aureobasidium* (Dothideales, Dothideomycetes), *Kondoa* (Agaricostilbales, Pucciniomycotina), *Neodevriesia* (Capnodiales, Dothideomycetes), *Phaeosphaeria* (Pleosporales, Dothideomycetes), *Pringsheimia*, and *Verrucocladosporium*. Such vertices formed an inter-connected core and expanded connections to the marginal vertices of the module (Figure 7C), suggesting that those taxa might be influential in the community assembly.

Comparison of the co-occurrence associations among networks of three plants showed that none of them was shared by all three hosts or by *T. aestivum* and *T. diccocides*. The co-occurrence between *Kondoa* and *Erythrobasidium* was common to *Ae. sharonensis* and *T. diccocides*, and the co-occurrence between *Crocicreas*—*Verrucocladosporium*—*Pringsheimia* was common to *T. aestivum* and *Ae. sharonensis* (Figure S2). Additionally, *Alternaria* (most abundant genus) showed a negative co-occurrence with *Vishniacozyma* in the *Ae. sharonensis* network.

### 3.6. Differentially Abundant Endophytes

**Figure 8:**
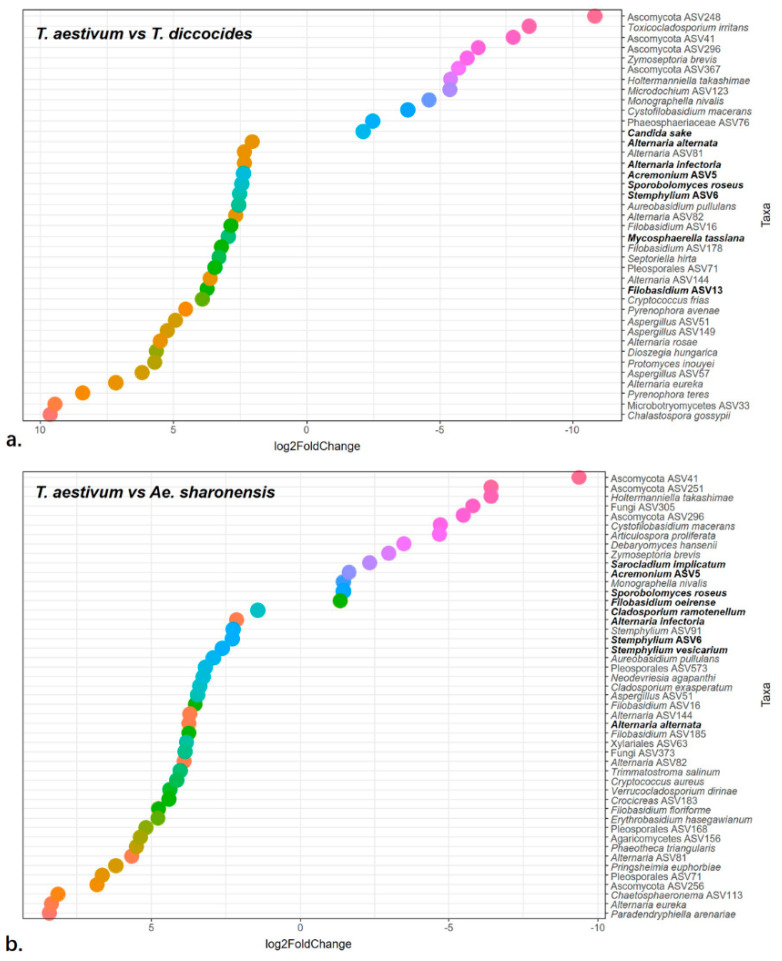
Differentially enriched fungal taxa. Differentially enriched taxa were identified using DeSEQ2 analysis. The graphs show a pair-wise comparison of fungal taxa that are differentially enriched at log2 fold or higher. Fungal taxa are shown on the Y-axis, and the dots represent significantly higher (Log_2_Fold > 0) and lower (Log_2_Fold < 0) abundances in wheat compared with either TD (**a**) or AS (**b**). Highly abundant taxa (see Figure 4) are in bold letters. Fungal genera are color coded.

Variance stabilizing transformation of all taxa was performed, in order to identify taxa that are differentially abundant in wheat compared with the two wild plant species (Tables S6 and S7). Comparison of wheat and *T. dicoccoides* revealed 12 fungal taxa, including the second most abundant species *C. sake,* that were significantly enriched (>log2 fold change) in wheat, and 27 taxa, including the highly abundant species *A. alternate*, *A. infectoria*, *Acremonium* ASV5, *Filobasidium* ASV13, *M. tassiana*, *S. roseus*, and *Stemphylium* ASV6, that were significantly enriched in *T. dicoccoides* (Figure 8a, Table S7). Compared with *Ae. sharonensis*, 14 fungal taxa including the abundant taxa *Acremonium* ASV5, *F. oeirense*, *S. implicatum*, and *S. roseus* were significantly enriched in *T. aestivum*, and 33 taxa, including the abundant taxa *A. infectoria*, *A. alternata*, *C. ramotenellum*, *Stemphylium* ASV6, *S. vesicarium* were significantly enriched in *Ae. sharonensis.* (Figure 8b, Table S7). A number of fungal taxa were significantly enriched in the two wild plant species compared with wheat, including some of the most highly abundant species, such as *A. infectoria, A. alternata*, *A. pullulans*, *Filobasidium* ASV16, and *Stemphylium* ASV6.

## 4. Discussion

Individual fungal endophytes can be used to enhance the performance of crop plants under agricultural conditions, e.g., by promoting growth and protecting plants from abiotic and biotic stresses [13,39]. Nevertheless, a combination of species (core community) rather than a single dominant species is assumed to contribute to optimal plants’ performance, especially under natural conditions [40]. Towards identification to individual and combinations of fungal endophytes with potential for wheat improvement, we generated profiles of fungal endophytes that are present in wheat and two wheat-related wild species. Comparative analysis of the FECs from the different plant species revealed that the wild species in each site contained a richer and more diverse repertoire of endophytic fungal taxa than wheat, however the combined communities of each plant species had similar diversity levels, suggesting that wheat plants in specific agricultural fields have microbiome-deficient status. The results also highlighted certain core fungal taxa that might play a role in community assembly and function.

A previous study of fungal endophytes in the same three plant species using culture-dependent approach recovered a total of 67 fungal OTUs, and between 2 to 4 OTUs per plant [41]. The culture-independent approach that we used in the current study revealed a much richer community, with an average number of 50 taxa per plant and up to 120 different taxa in a single plant. Using the ITS1f and ITS2 primers that capture a relatively wide range of fungal taxa [16,17], we uncovered a total of 665 fungal taxa that were divided between 159 genera. About 70% of the species were Ascomycota and the rest were divided between Basiomycota (21.50%), Mortierellomycota, Mucoromycota, and unassigned Fungi. This is in contrast to the culture-dependent study, in which 95% (64 out of 67) of the species were Ascomycota [41].

The most abundant genus in the entire population was *Alternaria*, of which *A. infectoria* and *A. alternata* were the predominant species. Two other highly abundant taxa were *Filobasidium* sp. and *C. sake*, both of which were not reported in the culture-dependent study. Notably, *C. sake* was the most abundant endophyte in bread wheat, even more abundant than *A. infectoria*, while it was much less abundant in the two wild species. While the reason for the differential abundance of *C. sake* and *A. infectoria* is unclear, it highlights the significant differences in the makeup of the FEC in cultivated and wild plant species. *A. infectoria* is associated with black point symptom or reduced grain quality [42,43], and has also been reported as an endophyte in non-wheat plants [44,45]. However, the very high incidence and abundance of *A. infectoria* in FECs from wheat-related wild plants that was observed in the current and a related study [2] argues that this fungus is part of the plants mycobiome rather than a pathogen.

The wild plant species exhibit greater diversity of FEC within an individual plant than wheat endophytes communities, indicating the potential influence of host genotype on within-community diversity. It is also possible that the farm management strategies result in lower species diversity in cultivated wheat compared with the wild species. Diversity difference in maize and soybean endophytes among field management strategies was demonstrated in [1], where the alpha diversity in soybean stems was higher in organic fields at a vegetative stage.

Interestingly, we did not find strong effects of the geographical location on the alpha diversity, which was nearly the same in each separate location for a given host species (Figure 5), especially for wheats. This is inconsistent with studies that revealed a greater effect of the geographical location than host genotype on microbial richness [46]. In addition, significant differentiation among FECs of local populations of the same species was clearly demonstrated. According to the model of additive partition of dispersion, the extent of such differentiation was larger to some degree for *T*. *aestivum*, whereas similar normalized values of effective number of local populations were obtained for all three host species. Lower values of alpha diversity (at all sites except Arsuf-Gaash), but larger differentiation extent in *T*. *aestivum* shows that the FECs from the three hosts produced close asymptotes in extrapolation for alpha-diversity, suggesting that the three plant species have similar potential capacities to accommodate diverse endophytic fungi.

Management practices seem to have a marginal effect on the structure of FECs in cultivated wheat [47]. Therefore, in addition to variable environmental conditions in wild habitats, the higher diversity and richness of mycobiota in wild plants might be attributed to the greater genetic diversity of wild plants populations, in particular when comparing with wheat populations in a specific agricultural field, which are genetically uniform. This notion is also congruent with previous reports on the importance of the host genotype in determining FEC composition in cereal phyllosphere [46]. Considering the lower diversity of wheat FECs in each location, wheat in agronomic environment might live in a microbiome-deficient status, and has the potential to accept additional (and functional) fungal symbionts.

While both wild and cultivated wheat species were dominated by species belonging to the genera *Alternaria*, *Candida*, *Filobasidium,* and *Cladosporium*, certain taxa were significantly differentially enriched, especially in the wild species compared to wheat. In addition to the greater genetic and environmental diversity, the greater richness of differentially abundant taxa in population of wild plants might also reflect the lack of agricultural treatments, in particular the use of fungicides. It is possible that at least some of the differentially enriched endophytes aid in deterring insects and pathogens; such taxa are expected to be reduced in wheat under agricultural conditions, in particular due to pesticide treatment. This possibility is corroborated in our analyses, wherein wheat cultivars from distinct biogeographic regions showed consistently fewer differentially abundant fungal taxa. FECs from wheat that were grown under organic mannagment were enriched for putative pathogenic species belonging to the genera *Zymoseptoria*, *Ceratobasidium*, and *Mycosphearella* compared with pesticide-treated wheat [47]. In contrast, the fungal taxa that were enriched in the wild plants in our study were primarily non-pathogenic compared to those from wheat, and are potentially involved in plant protection.

Cereal related grasses show high stochasticity in the assembly of endophytic microbiota [2,15]. Given the high stochasticity of microbial community assembly and the heterogeneous growth habitats of these plants, they are prone to accumulate highly heterogeneous microbiomes. In [9] was suggested that, when heterogeneity between samples is high, the recovered core microbiota would be limited and its composition will decline with an increasing number of samples. Accordingly, in our study, only a small proportion of the taxa might be considered candidates for core taxa. For example, we found 570 different fungal taxa in wheat, but only 105 were shared by all seven wheat populations, of which the functional roles and interaction with each other are mostly unknown (see Table S3 for detail).

Using network analysis we managed to explore the co-occurrence associations (namely, edges in a network) across geographical locations for certain hosts. However, the numbers of common associations dramatically decreased as more local communities were introduced, and no common association was shared by all communities (see Table S8 for detail). The associations shared by more than two communities (local networks) were extracted to generate a combined network of a certain host for network analysis. The results indicated that core taxa with high degrees and eigenvector centralities are not necessarily highly abundant in the community, as the most significant association were found between less abundant genera including *Crocicreas*, *Pringsheimia*, *Kondoa*, and *Verrucocladosporium*. In fact, the most abundant genera were largely absent in most networks. For example, *Alternaria* was absent from the *T. aestivum* and *T. diccocides* networks, and the only connection in *Ae. sharonensis* networks was a negative correlation to *Vishniacozyma*. Similarly, *C. sake*, which was the most abundant species in wheat, was absent from *T. diccocides* and *Ae. sharonensis* networks, and showed limited connection in the *T. aestivum* network. While not excluding the possible effect of the most abundant taxa on host performance, these results suggest that recruitment and assembly of the FECs are influenced by less abundant taxa, while the most highly abundant taxa have little or no effect on community assembly.

## 5. Conclusions

We have shown that individual FECs in wild plants relatives of wheat are richer and more diverse than in cultivated wheat. Assuming a nearly equal size of samples, the wild plants include a higher number of differentially abundant taxa than wheat, collectively supporting the notion that crop-related wild plant species represent a rich reservoir of potentially beneficial fungal endophytes. The fungal communities were characterized by high proportions of low abundant, sporadic taxa that probably have no functional role, and a subset of more highly abundant and prevalent (core) taxa with a possible functional role. Among the latter, a small number of taxa showed high level of association (co-occurrence), suggesting that they might represent hub taxa for recruitment of other taxa and assembly of the community. Functional analyses will be necessary in order to verify possible roles of putative candidate hub and functional taxa.

## Figures and Tables

**Table 1:** List of sampling locations. The numbers in parenthesis show: left, actual number of samples that were used in statistical analysis; right, total number of plants that were sampled in each site.

Title	Latitude	Longitude	Wild Plant Species	Wheat
Arsuf Ga’ash	32.22142	34.82036	*Aegilops sharonensis (29/44)*	*Triticum aestivum (37/42)*
Zikkim	31.61472	34.52537	*Aegilops sharonensis (41/42)*	─ ─ ─
Palmachim	31.93258	34.72893	*Aegilops sharonensis (37/40)*	*Triticum aestivum (42/43)*
Netanya	32.41755	34.88858	*Aegilops sharonensis (43/44)*	─ ─ ─
Almagor	32.90316	35.60003	*Triticum dicoccoides (34/42)*	*Triticum aestivum (30/43)*
Eliad	32.79048	35.74926	*Triticum dicoccoides (37/42)*	*Triticum aestivum (39/44)*
Ramot Menache	32.6035	35.06862	*Triticum dicoccoides (44/44)*	*Triticum aestivum (42/42)*
Karmiya	31.60292	34.54686	─ ─ ─	*Triticum aestivum (34/42)*
Hadera Elyachin	32.41429	34.91244	─ ─ ─	*Triticum aestivum (41/42)*

**Table 2:** Incidence-based interpolation and extrapolation curves indicating diversity in terms of the Hill numbers of order *q*: species richness (*q* = 0), exponential of Shannon entropy (*q* = 1) and inverse of Simpson concentration (*q* = 2), respectively.

Site	Diversity	Sample Size	Observed Diversity	Asymptotic Estimator
Almagor TA	*q* = 0	30	292	488.569
	*q* = 1	30	166.624	200.933
	*q* = 2	30	109.883	116.258
Eliad TA	*q* = 0	39	292	461.16
	*q* = 1	39	144.162	166.665
	*q* = 2	39	94.358	97.718
Ramot Menache TA	*q* = 0	42	301	454.842
	*q* = 1	42	146.45	170.794
	*q* = 2	42	88.982	92.284
Hadera Elyachin TA	*q* = 0	41	304	397.478
	*q* = 1	41	147.23	166.459
	*q* = 2	41	91.8	94.781
Arsuf Gaash TA	*q* = 0	37	316	483.901
	*q* = 1	37	165.651	191.492
	*q* = 2	37	108.939	113.407
Palmachim TA	*q* = 0	42	325	455.817
	*q* = 1	42	145.019	165.682
	*q* = 2	42	88.323	90.728
Karmiya TA	*q* = 0	34	303	457.091
	*q* = 1	34	153.679	180.549
	*q* = 2	34	95.708	99.516
Almagor TD	*q* = 0	34	351	439.704
	*q* = 1	34	190.752	212.173
	*q* = 2	34	128.701	133.344
Eliad TD	*q* = 0	37	393	548.44
	*q* = 1	37	206.382	236.2
	*q* = 2	37	130.888	136.023
Ramot Menache TD	*q* = 0	44	408	542.891
	*q* = 1	44	198.574	220.889
	*q* = 2	44	127.402	130.991
Netanya AS	*q* = 0	43	411	489.032
	*q* = 1	43	217.492	237.221
	*q* = 2	43	144.463	148.872
Arsuf Gaash AS	*q* = 0	29	290	404.916
	*q* = 1	29	166.35	194.229
	*q* = 2	29	110.598	116.609
Palmachim AS	*q* = 0	37	406	542.282
	*q* = 1	37	211.862	238.726
	*q* = 2	37	136.279	141.049
Zikkim AS	*q* = 0	41	395	535.214
	*q* = 1	41	204.404	227.225
	*q* = 2	41	134.336	138.495

**Table 3:** Variation within separate populations and species (α-diversity) for incidence data based on FECs of individual plants.

	Al ^a^	El	Ra	HE- Ne	AG	Pa	Ka- Zi	All Locations ^e^
AS ^b^				0.653	0.708	0.640	0.652	0.702 ^d^
TA	0.736 ^c^	0.673	0.698	0.656	0.699	0.608	0.655	0.734
TD	0.638	0.678	0.627					0.700

^a^ Locations are in columns: Almagor (Al), Arsuf Ga’ash (AG), Eliad (El), Hadera Elyachin (HE), Karmiya (Ka), Netanya (Ne), Palmachim (Pa), Ramot Menache (Ra), and Zikkim (Zi).^b^ Species are in rows: *Aegilops sharonensis* (AS), *Triticum aestivum* (TA) and *Triticum dicoccoides* (TD); ^c^ each entry in the first three rows and five columns equation (3) in [2] is the relative variation within a given species (row) in a given location (column); for example, 0.736 is the relative variation within species of TA in location Al. ^d^ Each entry in the last column, Equation (3) in [2] is the total relative variation within a given species (row) in all locations (entire set of the sampled plants of that species). For example, 0.702 is the total relative variation within species AS. ^e^ Pooled samples.

**Table 4:** Variation among populations in different locations (β-diversity) for incidence data, based on FECs of individual plants.

	AS ^d^	TA	TD
Number of FECs	4	7	3
*D* ^a^	0.125	0.190	0.151
${}_{\;}^{1}D\left(TM\right)$ ^b^	2.637	4.251	2.093
${}_{\;}^{1}nD\left(TM\right)$ ^c^	0.546	0.542	0.547

^a^ Extent of differentiation among populations of plants (represented by their FECs), based on the additive partition of dispersion; all differentiation estimates were proven to be significant (*p* < 0.001) with the permutation test (1000 random reshufflings), according to [33] and [29]. ^b^ Number of effectively different populations of FECs according to [34]. ^c^ Normalized number of effectively different populations of FECs [Equation (3) in [2]]. ^d^ Species encoding: Aegilops sharonensis (AS), Triticum aestivum (TA) and Triticum dicoccoides (TD).

**Table 5:** Variation among populations in different locations (β-diversity) for abundance Hellinger transformed data based on FECs of individual plants.

	AS ^d^	TA	TD
Number of FECs	4	7	3
*D* ^a^	0.314	0.367	0.288
${}_{\;}^{1}D\left(TM\right)$ ^b^	2.138	3.200	1.749
${}_{\;}^{1}nD\left(TM\right)$ ^c^	0.379	0.367	0.375

^a^ Extent of differentiation among populations of plants (represented by their FECs), based on the additive partition of dispersion; all differentiation estimates were proven to be significant (*p* < 0.001) with the permutation test (1000 random reshufflings), according to [33] and [29]. ^b^ Number of effectively different populations of FECs according to [34]. ^c^ Normalized number of effectively different populations of FECs [Equation (3) in [2]]. ^d^ Species encoding: *Aegilops sharonensis* (AS), *Triticum aestivum* (TA) and *Triticum dicoccoides* (TD).

## Supplementary Materials

The following are available online at https://www.mdpi.com/2309-608X/6/3/180/s1, Figure S1: Assessment of the effect of host species on the structure of FECs according to incidence data. The PCoA (A) and CAP (B) ordination based on Dice dissimilarity metric calculated from binary incidence data. ANOSIM shows significance of differentiation among FECs of different species. Figure S2: Co-occurrence associations (edges in a network graph) shared among combined networks of the three plant species. Only a few edges are shared between TA and AS, and between AS and TD, suggesting that core members of the FECs in three hosts might interact with each other in a more complex way. Table S1: List of ASVs, prevalence and abundance and their taxonomic assignment. Table S2: List of ASVs, prevalence and abundance and their taxonomic assignment following agglomeration. Table S3: List of filtered ASVs, prevalence and abundance and their taxonomic assignment used in diversity analyses. Table S4: Network properties of co-occurrence networks from the three hosts. Table S5: Vertex properties in networks from the three host. Table S6: Differentially enriched fungal endophytes in TA over TD. Table S7: Differentially enriched fungal endophytes in TA over AS. Table S8: Presence of co-occurrence associations for endophytic fungal genera in three hosts at each site.

## References

[1] Gdanetz K., Noel Z., Trail F. (2020). The phytobiomes of a three-crop rotation: Influence of land management, host, and plant organ on microbial diversity. BioRxiv.

[2] Sun X., Kosman E., Sharon O., Ezrati S., Sharon A. (2020). Significant host- and environment-dependent differentiation among highly sporadic fungal endophyte communities in cereal crops-related wild grasses. Environ. Microbiol..

[3] Gdanetz K., Benucci G.M.N., Pol N.V., Bonito G. (2017). CONSTAX: A tool for improved taxonomic resolution of environmental fungal ITS sequences. BMC Bioinform..

[4] Johnson L.J., Bonth A.C.M.D., Briggs L.R., Caradus J.R., Finch S.C., Fleetwood D.J., Fletcher L.R., Hume D.E., Johnson R.D., Popay A.J. (2013). The exploitation of epichloae endophytes for agricultural benefit. Fungal Divers..

[5] Johnson J.M., Alex T., Oelmüller R. (2014). *Piriformospora indica*: The versatile and multifunctional root endophytic fungus for enhanced yield and tolerance to biotic and abiotic stress in crop plants. J. Trop. Agric..

[6] Zapalski M.K. (2011). Is absence of proof a proof of absence? Comments on commensalism. Palaeogeogr. Palaeoclimatol. Palaeoecol..

[7] Hardoim P.R., Overbeek L.S.V., Berg G., Pirttilä A.M., Compant S., Campisano A., Döring M., Sessitsch A. (2015). The hidden world within plants: Ecological and evolutionary considerations for defining functioning of microbial endophytes. Microbiol. Mol. Biol. Rev..

[8] Shade A., Handelsman J. (2011). Beyond the Venn diagram: The hunt for a core microbiome. Environ. Microbiol..

[9] Vandenkoornhuyse P., Quaiser A., Duhamel M., Van A.L., Dufresne A. (2015). The importance of the microbiome of the plant holobiont. New Phytol..

[10] Toju H., Peay K.G., Yamamichi M., Narisawa K., Hiruma K., Naito K., Fukuda S., Ushio M., Nakaoka S., Onoda Y. (2018). Core microbiomes for sustainable agroecosystems. Nat. Plants.

[11] Agler M.T., Ruhe J., Kroll S., Morhenn C., Kim S.-T., Weigel D., Kemen E.M. (2016). Microbial hub taxa link host and abiotic factors to plant microbiome variation. PLoS Biol..

[12] Toju H., Tanabe A.S., Sato H. (2018). Network hubs in root-associated fungal metacommunities. Microbiome.

[13] Rodriguez R.J., White J.F., Arnold A.E., Redman R.S. (2009). Fungal endophytes: Diversity and functional roles. New Phytol..

[14] Banerjee S., Walder F., Büchi L., Meyer M., Held A.Y., Gattinger A., Keller T., Charles R., Heijden M.G.A.V.D. (2019). Agricultural intensification reduces microbial network complexity and the abundance of keystone taxa in roots. ISME J..

[15] Hassani M.A., Özkurt E., Franzenburg S., Stukenbrock E.H. (2020). Ecological assembly processes of the bacterial and fungal microbiota of wild and domesticated wheat species. Phytobiomes J..

[16] Gardes M., Bruns T.D. (1993). Its primers with enhanced specificity for basidiomycetes—Application to the identification of mycorrhizae and rusts. Mol. Ecol..

[17] Smith D.P., Peay K.G. (2014). Sequence depth, not PCR replication, improves ecological inference from next generation DNA sequencing. PLoS ONE.

[18] Bolyen E., Rideout J.R., Dillon M.R., Bokulich N.A., Abnet C.C., Al-Ghalith G.A., Alexander H., Alm E.J., Arumugam M., Asnicar F. (2019). Reproducible, interactive, scalable and extensible microbiome data science using QIIME 2. Nat. Biotechnol..

[19] Martin M. (2011). Cutadapt removes adapter sequences from high-throughput sequencing reads. EMBnet J..

[20] Callahan B.J., McMurdie P.J., Rosen M.J., Han A.W., Johnson A.J.A., Holmes S.P. (2016). DADA2: High-resolution sample inference from Illumina amplicon data. Nat. Methods.

[21] Kõljalg U., Nilsson R.H., Abarenkov K., Tedersoo L., Taylor A.F.S., Bahram M., Bates S.T., Bruns T.D., Bengtsson-Palme J., Callaghan T.M. (2013). Towards a unified paradigm for sequence-based identification of fungi. Mol. Ecol..

[22] Andersen K.S., Kirkegaard R.H., Karst S.M., Albertsen M. (2018). ampvis2: An R package to analyse and visualise 16S rRNA amplicon data. BioRxiv.

[23] Oksanen J., Blanchet F.G., Friendly M., Kindt R., Legendre P., McGlinn D., Minchin P.R., O’Hara R.B., Simpson G.L., Solymos P. vegan: Community Ecology Package. https://CRAN.R-project.org/package=vegan.

[24] Reese G.C., Wilson K.R., Flather C.H. (2014). Performance of species richness estimators across assemblage types and survey parameters. Global Ecol. Biogeogr..

[25] Chiu C.H., Chao A. (2016). Estimating and comparing microbial diversity in the presence of sequencing errors. PeerJ.

[26] Hsieh T.C., Ma K.H., Chao A. (2016). iNEXT: An R package for rarefaction and extrapolation of species diversity (Hill numbers). Methods Ecol. Evol..

[27] Bray J.R., Curtis J.T. (1957). An ordination of upland forest communities of southern Wisconsin. Ecol. Monogr..

[28] Dice L.R. (1945). Measures of the amount of ecologic association between species. Ecology.

[29] Kosman E. (2014). Measuring diversity: From individuals to populations. Eur. J. Plant Pathol..

[30] Kosman E. (1996). Difference and diversity of plant pathogen populations: A new approach for measuring. Phytopathology.

[31] Kosman E., Leonard K.J. (2007). Conceptual analysis of methods applied to assessment of diversity within and distance between populations with asexual or mixed mode of reproduction. New Phytol..

[32] Anderson M.J., Walsh D.C.I. (2013). PERMANOVA, ANOSIM, and the Mantel test in the face of heterogeneous dispersions: What null hypothesis are you testing?. Ecol. Monogr..

[33] Kosman E., Ben-Yehuda P., Manisterski J. (2014). Diversity of virulence phenotypes among annual populations of wheat leaf rust in Israel from 1993 to 2008. Plant. Pathol..

[34] Scheiner S.M., Kosman E., Presley S.J., Willig M.R. (2017). Decomposing functional diversity. Methods Ecol. Evol..

[35] Kosman E., Chen X., Dreiseitl A., McCallum B., Lebeda A., Ben-Yehuda P., Gultyaeva E., Manisterski J. (2019). Functional variation of plant-pathogen interactions: New concept and methods for virulence data analyses. Phytopathology.

[36] Love M.I., Huber W., Anders S. (2014). Moderated estimation of fold change and dispersion for RNA-seq data with DESeq2. Genome Biol..

[37] R_Core_Team R: A Language and Environment for Statistical Computing. https://www.R-project.org/.

[38] Berry D., Widder S. (2014). Deciphering microbial interactions and detecting keystone species with co-occurrence networks. Front. Microbiol..

[39] Arnold A.E., Mejía L.C., Kyllo D., Rojas E.I., Maynard Z., Robbins N., Herre E.A. (2003). Fungal endophytes limit pathogen damage in a tropical tree. Proc. Natl. Acad. Sci. USA.

[40] Berg G., Rybakova D., Fischer D., Cernava T., Vergès M.-C.C., Charles T., Chen X., Cocolin L., Eversole K., Corral G.H. (2020). Microbiome definition re-visited: Old concepts and new challenges. Microbiome.

[41] Ofek-Lalzar M., Gur Y., Ben-Moshe S., Sharon O., Kosman E., Mochli E., Sharon A. (2016). Diversity of fungal endophytes in recent and ancient wheat ancestors *Triticum dicoccoides* and *Aegilops sharonensis*. FEMS Microbiol. Ecol..

[42] Kosiak B., Torp M., Skjerve E., Andersen B. (2004). Alternaria and Fusarium in Norwegian grains of reduced quality—A matched pair sample study. Int. J. Food Microbiol..

[43] Perello A., Moreno M., Sisterna M. (2008). Alternaria infectoria species-group associated with black point of wheat in Argentina. Plant Pathol..

[44] Serdani M., Crous P.W., Holz G., Petrini O. (1998). Endophytic fungi associated with core rot of apples in South Africa, with specific reference to Alternaria species. Sydowia.

[45] Scholtysik A., Unterseher M., Otto P., Wirth C. (2013). Spatio-temporal dynamics of endophyte diversity in the canopy of European ash (Fraxinus excelsior). Mycol. Prog..

[46] Sapkota R., Knorr K., Jørgensen L.N., O’Hanlon K.A., Nicolaisen M. (2015). Host genotype is an important determinant of the cereal phyllosphere mycobiome. New Phytol..

[47] Gdanetz K., Trail F. (2017). The wheat microbiome under four management strategies, and potential for endophytes in disease protection. Phytobiomes.

