# Supplementary material for: Stem Endophytic Mycobiota in Wild and Domesticated Wheat: Structural Differences and Hidden Resources for Wheat Improvement
Source: J Fungi (Basel). 2020 Sep 18;6(3):180. doi: 10.3390/jof6030180 (PMC7557378; doi:10.3390/jof6030180)
Supplement: Supplementary file 1 [file jof-06-00180-s001.zip › Table 3.docx]

**Table 3.** Variation within separate populations and species (α-diversity) for incidence data based on FECs of individual plants.

|  | Al ^a^ | El | Ra | HE- Ne | AG | Pa | Ka- Zi | all locations ^e^ |
| --- | --- | --- | --- | --- | --- | --- | --- | --- |
| AS ^b^ |  |  |  | 0.653 | 0.708 | 0.640 | 0.652 | 0.702 ^d^ |
| TA | 0.736 ^c^ | 0.673 | 0.698 | 0.656 | 0.699 | 0.608 | 0.655 | 0.734 |
| TD | 0.638 | 0.678 | 0.627 |  |  |  |  | 0.700 |

^a^ Locations are in columns: Almagor (Al), Arsuf Ga'ash (AG), Eliad (El), Hadera Elyachin (HE), Karmiya (Ka), Netanya (Ne), Palmachim (Pa), Ramot Menache (Ra), and Zikkim (Zi).

^b^ Species are in rows: *Aegilops* *sharonensis* (AS), *Triticum* *aestivum* (TA) and *Triticum* *dicoccoides* (TD);

^c^ each entry in the first three rows and five columns [${}^{1}{nD\left( T,KW \right)}$, eq. 3 in Sun et al. (2020)] is the relative variation within a given species (row) in a given location (column); for example, 0.736 is the relative variation within species TA in location Al.

^d^ Each entry in the last column [${}^{1}{nD\left( T,KW \right)}$, eq. 3 in Sun et al. (2020)] is the total relative variation within a given species (row) in all locations (entire set of the sampled plants of that species). For example, 0.702 is the total relative variation within species AS.

^e^ Pooled samples.
