# Supplementary material for: Stem Endophytic Mycobiota in Wild and Domesticated Wheat: Structural Differences and Hidden Resources for Wheat Improvement
Source: J Fungi (Basel). 2020 Sep 18;6(3):180. doi: 10.3390/jof6030180 (PMC7557378; doi:10.3390/jof6030180)
Supplement: Supplementary file 1 [file jof-06-00180-s001.zip › Table 4.docx]

**Table 4.** Variation among populations in different locations (β-diversity) for incidence data based on FECs of individual plants.

|  | AS ^d^ | TA | TD |
| --- | --- | --- | --- |
| # of FECs | 4 | 7 | 3 |
| *D* ^a^ | 0.125 | 0.190 | 0.151 |
| ${}^{1}{D(TM)}$ ^b^ | 2.637 | 4.251 | 2.093 |
| ${}^{1}{nD(TM)}$ ^c^ | 0.546 | 0.542 | 0.547 |

^a^ Extent of differentiation among populations of plants (represented by their FECs) based on the additive partition of dispersion; all differentiation estimates were proven to be significant (*p* < 0.001) with the permutation test (1000 random reshufflings) according to Kosman et al. (2014, p. 565) and Kosman (2014, pp. 475-476).

^b^ Number of effectively different populations of FECs according to Scheiner et al. (2017).

^c^ Normalized number of effectively different populations of FECs [eq. 3 in Sun et al. (2020)].

^d^ Species encoding: *Aegilops* *sharonensis* (AS), *Triticum* *aestivum* (TA) and *Triticum* *dicoccoides* (TD).
