# Supplementary figures and images for: Stem Endophytic Mycobiota in Wild and Domesticated Wheat: Structural Differences and Hidden Resources for Wheat Improvement
Source: J Fungi (Basel). 2020 Sep 18;6(3):180. doi: 10.3390/jof6030180 (PMC7557378; doi:10.3390/jof6030180)

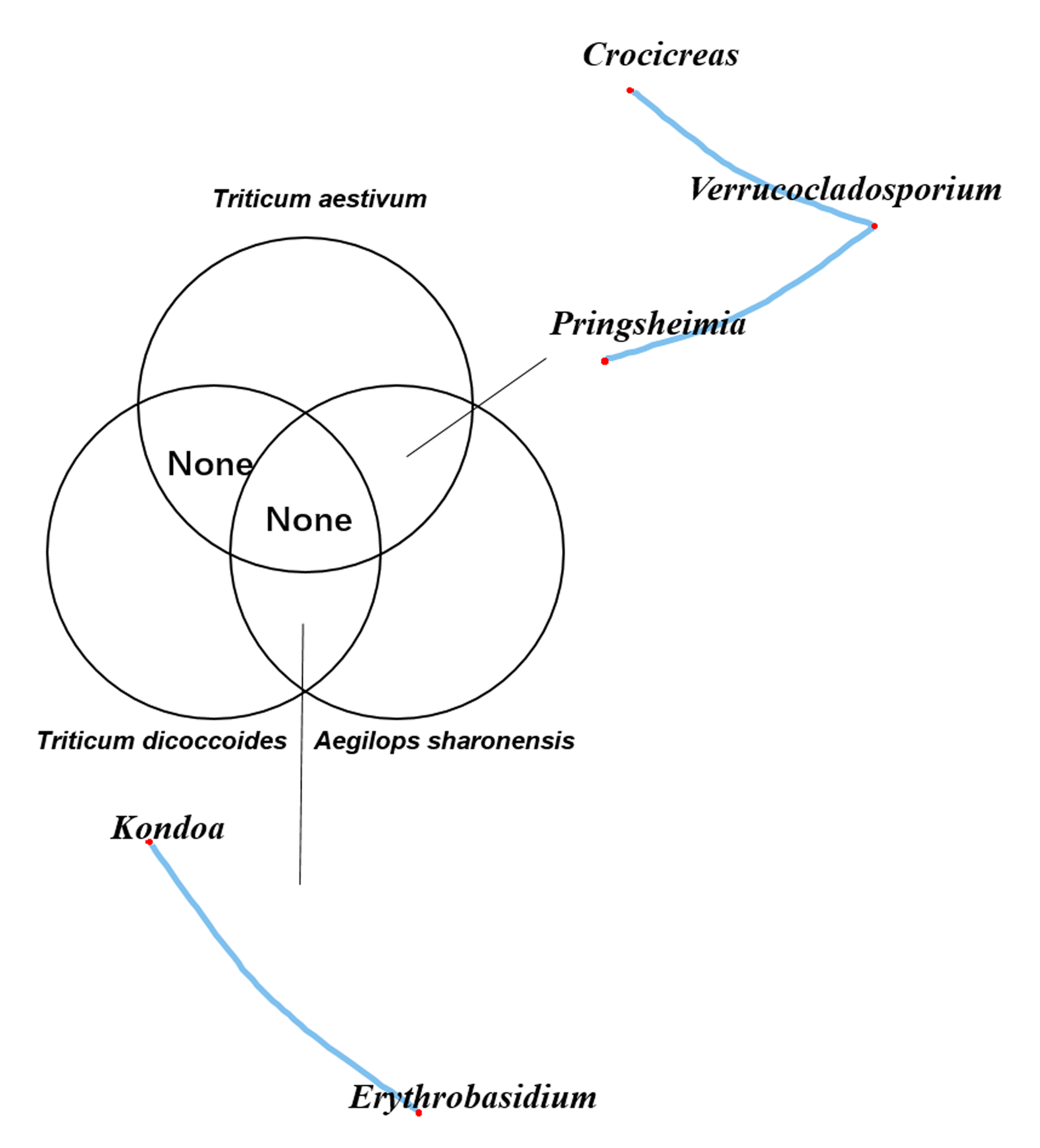

Supplement: Supplementary file 1 [file jof-06-00180-s001.zip › Figure S2.tif]
